# Axial Compression Performance of Square Thin Walled Concrete-Filled Steel Tube Stub Columns with Reinforcement Stiffener under Constant High-Temperature

**DOI:** 10.3390/ma12071098

**Published:** 2019-04-02

**Authors:** Xuetao Lyu, Yang Xu, Qian Xu, Yang Yu

**Affiliations:** 1 Transportation and Civil Buildings College, Foshan University, Foshan 528000, China; 2 College of Civil Engineering, Liaoning Technical University, Fuxin 123000, China; xuyang5213@gmail.com (Y.X.); qiang.xu336@gmail.com (Q.X.); 3 School of Civil and Environmental Engineering, University of Technology Sydney, Sydney, NSW 2007, Australia

**Keywords:** thin-walled square steel tubular concrete, stub column, reinforcement stiffener, high-temperature, mechanical behavior

## Abstract

This study investigated the axial compressive performance of six thin-walled concrete-filled steel tube (CFST) square column specimens with steel bar stiffeners and two non-stiffened specimens at constant temperatures of 20 °C, 100 °C, 200 °C, 400 °C, 600 °C and 800 °C. The mechanical properties of the specimens at different temperatures were analyzed in terms of the ultimate bearing capacity, failure mode, and load–displacement curve. The experiment results show that at high temperature, even though the mechanical properties of the specimens declined, leading to a decrease of the ultimate bearing capacity, the ductility and deformation capacity of the specimens improved inversely. Based on finite element software ABAQUS, numerical models were developed to calculate both temperature and mechanical fields, the results of which were in good agreement with experimental results. Then, the stress mechanism of eight specimens was analyzed using established numerical models. The analysis results show that with the increase of temperature, the longitudinal stress gradient of the concrete in the specimen column increases while the stress value decreases. The lateral restraint of the stiffeners is capable of restraining the steel outer buckling and enhancing the restraint effect on the concrete.

## 1. Introduction

The thin-walled concrete-filled steel tubular (CFST) column is designed based on the general CFST column [1,2,3], in which the thickness of the steel tube is reduced. This type of component can reduce the project cost while inheriting the superior performance of ordinary CFST columns. This satisfies the bearing capacity requirements in middle and small high-rise buildings. Due to perfect performance in bearing capacity, plasticity, and ductility, CFST columns have been widely applied in engineering practice. Compared with a circular cross-section, a square one is more commonly used, gaining greater moment resistance and stability efficiency [4]. However, because of the unevenly and weaker confinement, CFST columns with a square cross-section have a relatively smaller bearing capacity and ductility, while the steel tube is more prone to buckling [5,6].

To improve the mechanical properties of square or rectangular CFST columns, the key issue is to increase the constraint of the steel tube on concrete. Aiming at achieving this objective, many researchers have proposed different construction strategies, including setting up the stiffened straight rib and the oblique rib [7,8,9], the tensile sheet [10], the binding bar [11], the corner brace [12], etc. The results showed that these enhanced measures can delay the buckling of steel tubes and thus improve the bearing capacity and ductility of the columns. Besides, timber is considered as the infill element to put into the CFST to reduce the structural weight and improve the ductility, capacity, and energy dissipation [13].

To date, the research in this field has focused on the comprehensive static and seismic behaviors of such a kind of composite column, as well as experimental investigations into structural behavior of composite columns subjected to axial [14,15], eccentric [16,17], and hysteretic loads [18,19,20], analytical studies on buckling of steel tubes [11,21] and the stress–strain relationship model of concrete [22,23]. However, to the best knowledge of the authors, the numerical and experimental studies on the responses of short square and rectangular CFST columns exposed to fire have been rarely reported.

Consequently, this paper studies the mechanical properties of thin-walled square steel tubular concrete columns under high temperature via both experimental and numerical investigations. The effects of different temperature fields and settings of stiffening ribs on the mechanical properties and failure modes of the specimens are investigated. The test results are presented in terms of load–displacement curves. Moreover, the stress distributions of concrete, steel tube, and reinforcement stiffener of each specimen are analyzed using finite element software ABAQUS v6.16. The outcome of this research is capable of providing a guide for the design of such a kind of component in practical engineering and post-disaster restoration.

## 2. Experimental Study

### 2.1. Design and Fabrication of Specimens

In this study, eight thin-walled square CFST short columns were designed and fabricated, in which six specimens were stiffened and two specimens not stiffened. The width of the steel tube was 160 mm, the wall thickness of the steel tube 2 mm, and the height of the test piece 480 mm. One specimen was tested under ambient temperature while the remaining seven specimens were heated to 100 °C, 200 °C, 400 °C, 600 °C and 800 °C, respectively. The detailed information about the specimens is given in Table 1. The grade of the concrete is C30, the grade of the steel tube is Q235, and the grade of the steel bar is HPB300. The properties of steel and concrete materials were obtained according to the room-temperature tensile test method (GB/T228-2002) and the normal concrete mechanical properties testing method standard (GB/T50081-2002), as shown in Table 2 and Table 3. 

**Figure 1:**
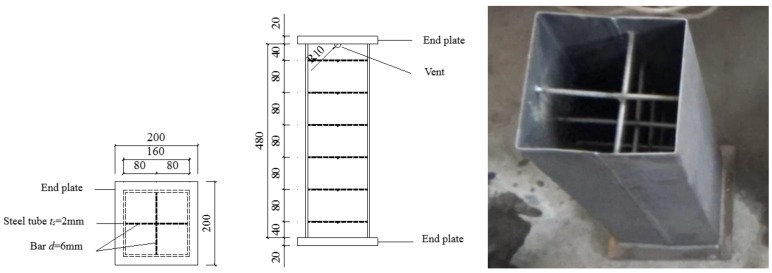
Design of short column specimen.

The design of test specimens is shown in Figure 1. The steel sheet with the thickness of 2 mm was cut and bent into a U-shaped plate. Then, two U-shaped plates were spliced and welded together by the butt weld. The steel stiffeners are composed of HPB300 hot-rolled round steel bars, and the steel bars were welded to the inner wall of the steel tube. A semi-circular venting hole with a diameter of 20 mm was designed at the upper end of the steel tube to facilitate evaporation and discharge of moisture inside the concrete of the specimen under high temperature. Two steel plates with the dimensions of 200 mm × 200 mm × 20 mm were designed as the cover plates of the test specimen, one of which was selected to be welded to one end of the test specimen as the bottom plate. After pouring the concrete, the other cover plate was taken to be welded to the other end of the test specimen as the roof plate.

### 2.2. Testing Setup

**Figure 2:**
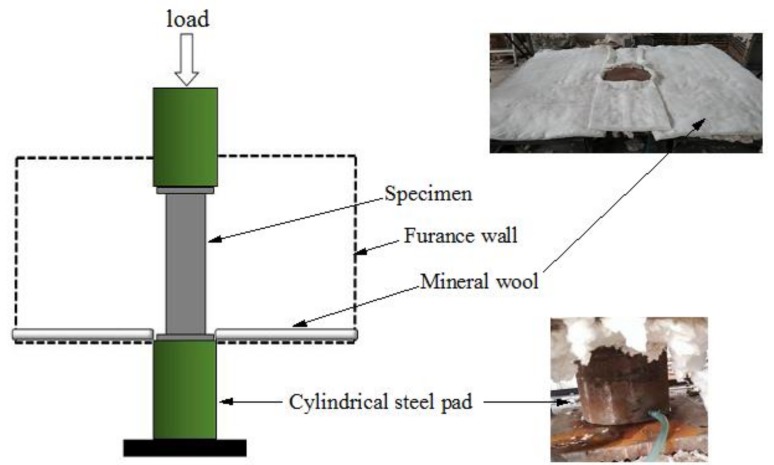
Schematic of the loading device.

The experimental tests were conducted at the Structure Experiment Center in Xiangtan University, China. The specimens are heated in an electric furnace which has six sets of independent temperature control system with intelligent controllers (Series No. 2100, Xiangtan Sanxing Instrument Co Ltd., Xiangtan, China). The loading device is a two-channel electro-hydraulic servo actuator with a rated loading capacity of 4000 kN. Data collection is implemented by a multi-channel coordinated load data acquisition system equipped with M400 temperature data acquisition and management software. The total height of the specimen is 520 mm (including the thickness of the cover plate), and the height of the furnace is 630 mm. To ensure the specimen is placed in the middle location of the furnace, two plates with specific heights are put at both ends of the specimen. Meanwhile, a water-cooled head is established on the plates at both ends to avoid the large deformation caused by the high temperature of the plate in the test. The schematic of the loading device is shown in Figure 2. The plates, water-cooled head, specimen, and loading plate of the loading device are aligned. Fire-resistant cotton is employed to guarantee that the temperature in the furnace will not leak during the testing. Finally, the experimental furnace body is assembled.

### 2.3. Test Process

**Figure 3:**
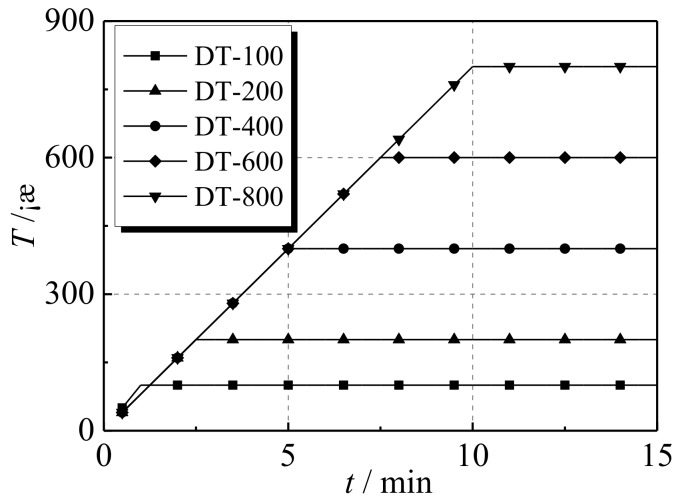
Heating curves of the specimens.

After the pre-heating test, eight specimens were heated to the required temperatures as shown in Table 4, and the temperature was kept at a constant level for three hours. The heating curves of the specimens are displayed in Figure 3. In this way, the temperature of each section of the specimen could steadily reach the specified value. Under constant temperature, the axial loading of the specimen is based on displacement control, which is implemented by the test machine. The target displacement was set as 20 mm and the loading rate set as 1.0 mm/min. When the decrease phenomenon was observed in the displacement–load curve of the specimen, the loading rate was increased to 2.0 mm/min so that the drop rate of the loading plate could keep pace with the displacement change rate of the specimen in the descending section of displacement–load curve. Accordingly, more reliable testing data can be obtained from the experiment.

### 2.4. Experimental Results and Failure Modes

**Figure 4:**
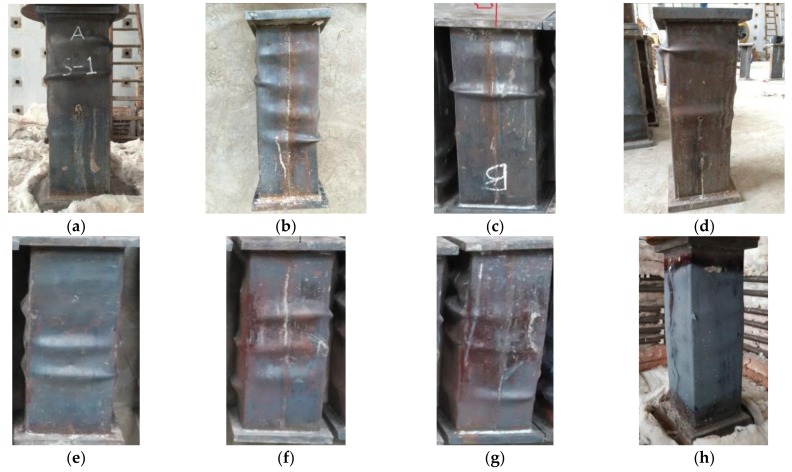
View of specimens after test (**a**) DS-20; (**b**) DS-100; (**c**) DS-200; (**d**) DS-200-0; (**e**) DS-400; (**f**) DS-600; (**g**) DS-600-0; (**h**) DS-800.

Figure 4 shows the photos of the final appearances of specimens after the experimental tests. It can be seen that the steel tubes exhibit buckling (directional deviation from concrete) after the tests of high-temperature 20–200 °C. The locations of the buckling at different sides of the specimen are not the same. The buckling mainly occurs in the middle or at the ends of the specimen, which is asymmetrically distributed on the opposite sides. The curvatures of buckling were found to be similar after measurements. Among them, the curvature degree of steel tube buckling of the non-stiffened specimen (DS-200-0) was high. The rupture phenomenon happens due to the buckling at the top area of the steel tube, leading to slight bending of the whole specimen. It is of note that its integrity is not as good as that of rebar stiffened specimens. The buckling is mainly in the middle area of the steel tube for the specimens exposed to high temperature of 400–600 °C. The steel tube buckling of the case of 800 °C high temperature mainly occurred in the adjacent position of the central steel tube. Due to the fact that the outer surface of the steel tube is covered by an oxide layer, the buckling curvature was not as sharp as that of the specimen under low temperature.

**Figure 5:**
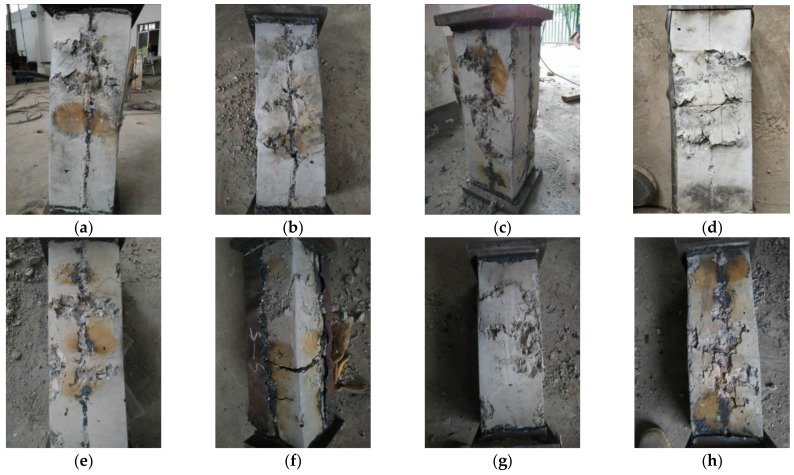
View of specimens after opening steel tubes (**a**) DS-20; (**b**) DS-100; (**c**) DS-200; (**d**) DS-200-0; (**e**) DS-400; (**f**) DS-600; (**g**) DS-600-0; (**h**) DS-800.

Figure 5 shows the photos of the specimens after they are split. It is obvious to observe that most of the concrete is not damaged except the concrete at the oblique line region at the opposite sides of the steel tube buckling location, where serious damage occurs. It is also found that there are visible multiple shear slip lines at the side of the specimen, forming shear surface (dip angle around 45°). Only a few small cracks occur along the direction of concrete fracture. Since the concrete is still hard and not easy to break, the damage type of each specimen is shear failure. Among them, the outer surface of the concrete of the specimen at 800 °C temperature shows a certain degree of color change. A broken oblique section is located around the inner part of the specimen, and the shear failure surface cracks are small in large number.

### 2.5. Analysis of Test Results

**Figure 6:**
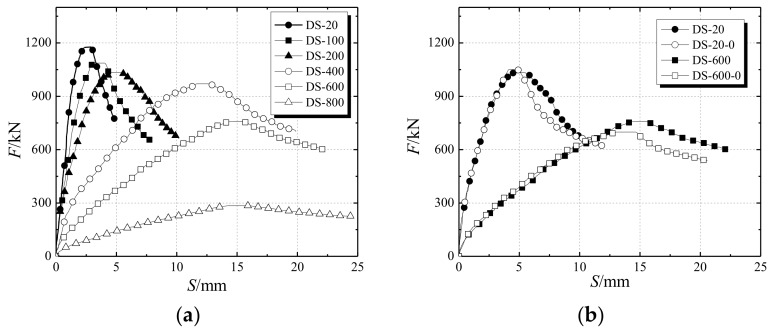
Load–displacement diagram (**a**) effects of different temperatures (**b**) effects of stiffening rib of steel bar.

Figure 6a describes the load–displacement curves of the specimens under different temperature effects. The slope of the load–displacement curve of each specimen decreases with the increase of temperature while the peak displacement corresponding to the ultimate bearing capacity increases. The elastic modulus of the specimen under high temperature decreases so that the ductility of the specimen is improved. The ultimate bearing capacity of each specimen shows a significant decreasing trend with the increase of temperature. 

Figure 6b demonstrates the effect of the stiffening rib on the ultimate bearing capacity of the specimen under high temperature conditions. For the specimens with 200 °C temperature, the ultimate bearing capacities of the two types of specimen (stiffened and non-stiffened) are almost the same. It can be seen that the steel stiffened ribs have a minor effect on the ultimate bearing capacity. The load–displacement curve of the DS-200 (steel bar stiffened specimen), however, is relatively flat, showing good plasticity and ductility. For the specimens at 600 °C high-temperature, the ultimate bearing capacity of DS-600 is 73 kN higher than that of DS-600-0 (non-stiffened specimen). Setting stiffening ribs for the specimens under high temperature increases the ultimate bearing capacity of the specimen to a certain extent. In addition, the load–displacement curve of DS-600-0 is steep on both sides of the ultimate load, which indicates that the stiffening rib can still improve the plasticity and ductility of the specimen.

**Figure 7:**
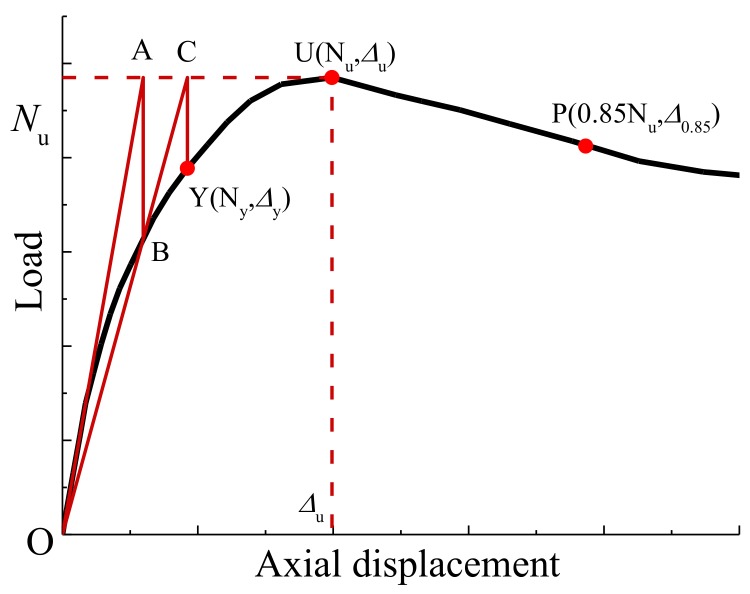
The geometric drawing method.

Table 5 summarizes the testing results of compressive stiffness (EA), yield load (*N_y_*), yield displacement (*△*_y_), peak load (i.e. residual bearing capacity *N_u_* after fire exposure), peak displacement (*△*u), and 0.85 *Nu* after peak load (*△*0.85) and ductility coefficient (*μ*_△_ with the expression in Equation (1)). The geometric drawing method was employed to find the yield load and the yield displacement of the load–displacement curve by making a characteristic line, as shown in Figure 7. First, the peak tangent U was used as the horizontal tangent of the curve, intersecting the origin tangent (EA) at point A, passing the point A as the vertical axis of the displacement axis, intersecting the curve at point B, connecting the line OB to the line AU at point C, and passing the point C. The vertical line of the displacement axis intersects the point Y, which is the yield point. Hence, the horizontal and vertical coordinates Δ*y* and *N_y_* corresponding to the Y point are the yield displacement and the yield load, respectively.

(1) $$\mu_{△}=\frac{{△}_{0.85}}{{△}_{u}},$$

It can be seen from Table 5 that the residual bearing capacity and compressive stiffness of the test specimens are significantly reduced when the temperature of the furnace is increased. For example, compared with the properties of the specimen at normal room temperature, the bearing capacities of the specimens at temperatures of 100 °C, 200 °C, 400 °C, 600 °C and 800 °C decrease 7.72%, 12.3%, 17.64%, 35.55%, 75.83%, respectively. The compressive stiffness properties of these specimens is reduced by 11.79%, 14.83%, 63.12%, 72.24% and 90.49%, respectively. Compared to the bearing capacity, the compressive stiffness is more affected by the high temperature. It can be seen that the critical temperature of the test specimen is between 200 °C and 400 °C, which indicates that the decrease rates of residual bearing capacity and compressive stiffness of the test specimen increase after the temperature exceeds 200 °C, which is mainly due to the fact that after the furnace temperature exceeds the critical temperature, the strength of the concrete is rapidly decreased. The ductility coefficient generally decreases with the increase of the temperature of the furnace. Except for the DS-20 and DS-400 with low ductility coefficients, the overall trend is in agreement with the conclusion.

Compared with the specimens without stiffening steel bars, the ultimate bearing capacities of the specimens with steel stiffeners at temperatures of 200 °C and 600 °C are changed by −1.71% and 9.05% respectively. Similarly, the compressive stiffness of the specimens with steel stiffeners is slightly reduced, but their ductility coefficients are not changed. Overall, the setting of stiffened steel bars has a positive effect on improving the mechanical properties of CFST columns.

## 3. Numerical Analysis of Finite Field Model

### 3.1. Model Establishment

#### 3.1.1. Temperature Field Model

For the temperature field model, the concrete adopts the eight-node three-dimensional solid thermal Analysis Unit DC3D8. The outer steel tube adopts the shell thermal analysis Unit S4R, and the inner steel bar is T3D2 by the one-dimensional truss thermal analysis unit.

The thermal performance model of steel and calcareous concrete was suggested by Lie and Irwin [24]. The moisture inside the concrete evaporates around 100 °C and the heat is absorbed in the meantime, which has a certain effect on the temperature distribution of the cross-section of the specimen. Therefore, according to the method adopted in ref. [24], the percentage of water content in concrete before evaporation is assumed to be 5%. The thermal model of concrete is modified to take the water effect into account.

In the furnace, the heat that transmits from the air to the component is mainly radiation and convection, while the heat that transmits inside the component is heat conduction. The temperature of the cross-section of the component continuously changes over time, which belongs to transient heat transfer. According to Fourier’s law and the conservation law of energy, the distribution of temperature at the cross-section of the component can be obtained via solving the heat conduction differential equation of the cross-section.

The initial temperature is set to be 20 °C. For the convection and radiation, the comprehensive emissivity is set as 0.5 and the convective heat transfer coefficient at the outer surface of the steel tube is set as 25 W/(m^2^·°C) [25]. The interaction between the concrete and the inner surface of the steel tube is determined by the interaction. The means of heat transmission is heat conduction. Surface-to-surface contact is adopted. The outside surface of the concrete is the main surface, and the inner surface of the steel tube is the secondary surface. Due to the shrinkage deformation of the concrete during the curing process of the specimen, the interface between the steel tube and the concrete has a gap, and a series of physical and chemical reactions occur inside the specimen at high temperature, which changes the compositions, contents, and states of the media at the interface, and is sometimes accompanied by water migration. In this study, the contact thermal resistance of the steel tube and concrete in the CFST columns is set as 0.01 (m^2^·°C)/W.

The contact type among the steel tube, concrete and the stiffener is set to be “Tie” contact. Due to the heat transfer process inside the main simulated components, the constraints between the components is simplified, and the thermal resistance between the concrete shrinkage and the steel bars is ignored.

**Figure 8:**
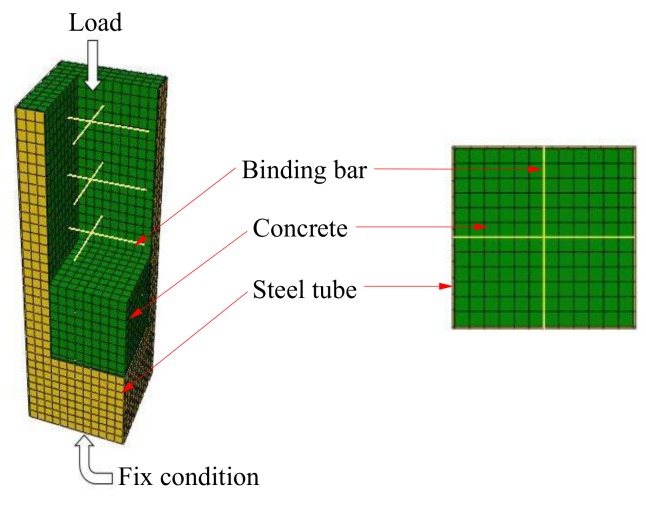
Boundary conditions and element mesh.

According to the default rule, the steel bar is meshed into a line unit length of 16 mm. Using the structural networking method, the concrete is meshed into a hexahedral unit with a side length of 16 mm. The steel tube parts are meshed into quadrilateral elements with the same side length. The boundary conditions and element mesh are shown in Figure 8.

#### 3.1.2. Mechanical Field Model

In the analysis process, the steel tube adopts the shell element S4R while the concrete adopts the eight-node reduction integral three-dimensional solid element C3D8R. The steel bar selects the two-node one-dimensional linear truss element T3D2.

The models used for the stress–strain relationship of the steel under high temperature and the free expansion strain of the steel under high temperature were suggested by Lie [26]. Due to a short furnace duration, the creep at high temperature of the steel and concrete was not considered in this study. The expression for transient thermal strain of the concrete was suggested by Guo and Shi [27]. The model for the tensile stress–strain relationship of the concrete was suggested by Hong [28]. The relevant thermodynamic formulae of the materials are given as follows.

(1) Mechanical properties of the steel under high temperature

1) Stress–strain relationship

(2) $$\sigma_{s}=\left\{\begin{array}{ll} \frac{f\left(T,0.001\right)}{0.001}\varepsilon_{s\sigma } & \varepsilon_{s\sigma }\leq \varepsilon_{p}\\ \frac{f\left(T,0.001\right)}{0.001}\varepsilon_{p}+f\left[T,\left(\varepsilon_{s\sigma }-\varepsilon_{p}+0.001\right)\right]-f\left(T,0.001\right) & \varepsilon_{s\sigma }>\varepsilon_{p} \end{array}\right.,$$

where σ_s_ denotes the steel stress, *ε*_sσ_ denotes the steel strain, *ε*_p_ denotes the steel strain corresponding to maximum stress, and *T* denotes the temperature. In this study, $\varepsilon_{p}=4\times 10^{-6}f_{y}$, $f\left(T,0.001\right)=\left(50-0.04T\right)\times \left[1-\exp\left(-30+0.03T\right)\sqrt{0.001}\right]\times 6.9$, and $f\left[T,\left(\varepsilon_{s\sigma }-\varepsilon_{p}+0.001\right)]=\left(50-0.04T\right)\times [1-\exp\left(-30+0.03T\right)\sqrt{\varepsilon_{s\sigma }-\varepsilon_{p}+0.001}\right]\times 6.9$. 

2) Thermal free expansion

(3) $$\varepsilon_{\mathrm{sth}}=\left\{\begin{array}{ll} (0.004T+12)T\times 10^{-6} & T<1000{}^{\circ}C\\ 16T\times 10^{-6} & T\geq 1000{}^{\circ}C \end{array}\right.,$$

where *ε*_sth_ denotes free expansion strain of the steel.

(2) Mechanical properties of the concrete under high-temperature

1) Stress–strain relationship in compression

(4) $$\sigma_{c}=\left\{\begin{array}{cc} f_{c}^{\prime }(T)\left[1-{\left(\frac{\varepsilon_{\max}-\varepsilon_{c\sigma }}{\varepsilon_{\max}}\right)}^{2}\right] & \varepsilon_{c\sigma }\leq \varepsilon_{\max}^{}\\ f_{c}^{\prime }(T)\left[1-{\left(\frac{\varepsilon_{c\sigma }-\varepsilon_{\max}}{3\varepsilon_{\max}}\right)}^{2}\right] & \varepsilon_{c\sigma }>\varepsilon_{\max}^{} \end{array}\right.,$$

where σ_c_ denotes the concrete stress, *ε*_cσ_ denotes the concrete strain, and *ε*_max_ denotes the concrete strain corresponding to maximum stress. In this study, $\varepsilon_{\max}=0.0025+\left(6T+0.04T^{2}\right)\times 10^{-6}$. $f_{c}^{\prime }\left(T\right)$ denotes the compressive strength of concrete at temperature *T*, and its mathematical expression is given as follows.

(5) $$f_{c}^{\prime }(T)=\left\{\begin{array}{cc} f_{c}^{\prime } & 0{}^{\circ}C<T<450{}^{\circ}C\\ f_{c}^{\prime }\left[2.011-2.353\left(\frac{T-20}{1000}\right)\right] & 450{}^{\circ}C\leq T\leq 874{}^{\circ}C\\ 0 & T>874{}^{\circ}C \end{array}\right.,$$

2) Thermal free expansion of the concrete

(6) $$\varepsilon_{\mathrm{th}}=(0.008T+6)T\times 10^{-6},$$

where *ε*_th_ denotes free expansion strain of the concrete.

3) Thermal transient strain of the concrete

(7) $$\varepsilon_{\mathrm{ctr}}=\frac{\sigma_{c}}{f_{c}}\left[72{\left(\frac{T}{1000}\right)}^{2}-\left(\frac{T}{1000}\right)\right]\times 10^{-3},$$

where *ε*_ctr_ denotes transient strain of the concrete.

4) Stress–strain relationship in tension

(8) $$\sigma_{\mathrm{ct}}=\left\{\begin{array}{l} E_{c}\left(T\right)\varepsilon \;\;\;\;\;\;\;\;\;\varepsilon <\varepsilon_{\mathrm{cr}}\\ f_{t}{}^{\prime }\left(T\right)-0.1f_{t}{}^{\prime }\left(T\right)\frac{\varepsilon -\varepsilon_{\mathrm{cr}}}{\varepsilon_{\mathrm{cr}}}\;\;\;\varepsilon_{\mathrm{cr}}<\varepsilon \leq 2\varepsilon_{\mathrm{cr}}\\ 0.9f_{t}{}^{\prime }\left(T\right)\;\;\;\;\;\;\;\;\;\varepsilon \leq 2\varepsilon_{\mathrm{cr}} \end{array}\right.,$$

where σ_ct_ denotes the concrete stress in tension, *ε* denotes the tensile strain of the concrete, *ε*_cr_ denotes the concrete tensile strain corresponding to maximum stress, *E_c_* denotes the elastic modulus of the concrete, and $f_{t}^{\prime }$ denotes axial tensile strength. Here, $f_{t}^{\prime }=0.09f_{c}^{\prime }$ and $\varepsilon_{cr}=f_{t}^{\prime }\left(T\right)/E_{c}\left(T\right)$.

The contact relationships among the steel tube, the rebar, and the concrete are given as follows:

(1) The contacts between inner surfaces of the steel hollow section and the concrete are set as surface-to-surface contact. The normal contact property is general hard contact while the tangential contact property is defined by friction. The interfacial friction coefficient is set as 0.35 [29].
(2) The concrete and the rebar are set as embedding constraints.
(3) The binding constraint tie is set between the steel tube and the steel bar.

The boundary conditions at both ends of the member are configured as follows. Translational degrees of freedom and rotational freedom of the bottom end are fixed as well as the horizontal translational degrees of the top end. The three-direction rotational degrees of the top end are set as default values. In addition, the displacement U3 is set according to real test conditions.

The mesh division of the mechanical field model is consistent with the temperature field model with 16 mm unit length to ensure the node temperature calculation accurately.

### 3.2. Validation of Finite Element Models

**Figure 9:**
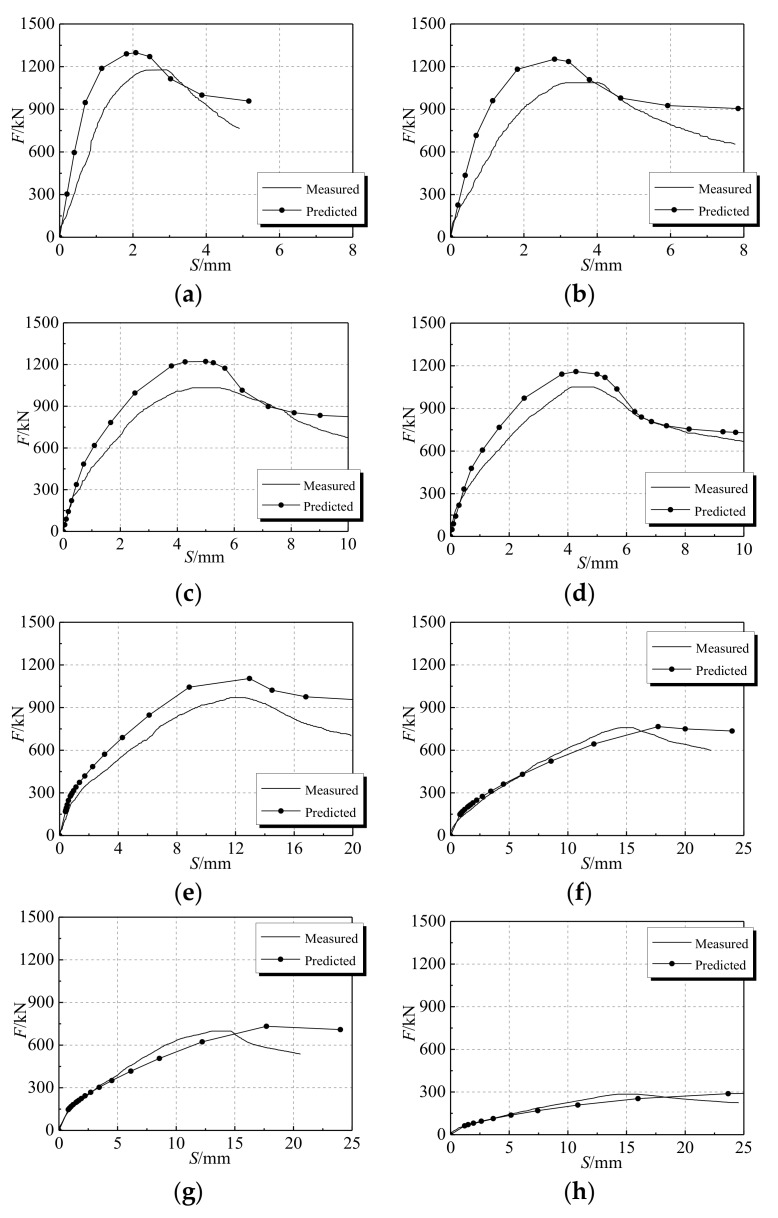
Load–displacement of experiment and numerical simulation (**a**) DS-20; (**b**) DS-100; (**c**) DS-200; (**d**) DS-200-0; (**e**) DS-400; (**f**) DS-600; (**g**) DS-600-0; (**h**) DS-800.

Based on the calculation model of the constant temperature under axial pressure in the earlier section, the capacity of the model was validated by comparing the numerical results with the corresponding experimental results, which are illustrated in Figure 9. It is noticeable that there are differences between the experimental results and simulation results. The main reason leading to this phenomenon is that the finite element method (FEM) simulation is carried out under perfect conditions, while various non-perfect conditions exist in the practical case. For instance, concrete pouring does not give a dense structure, and a certain gap between the steel tube and the concrete leads to geometric imperfections. Furthermore, the inherent error of the loading device and the linear variable differential transformer (LVDT, displacement sensor) also can cause deviation between the experimental and numerical results. The mechanical property errors are caused by chemical and physical reactions inside the specimen at high temperature. Since the errors are comparatively small and in the controllable range, the numerical simulation results are still reliable with a high degree of confidence.

**Figure 10:**
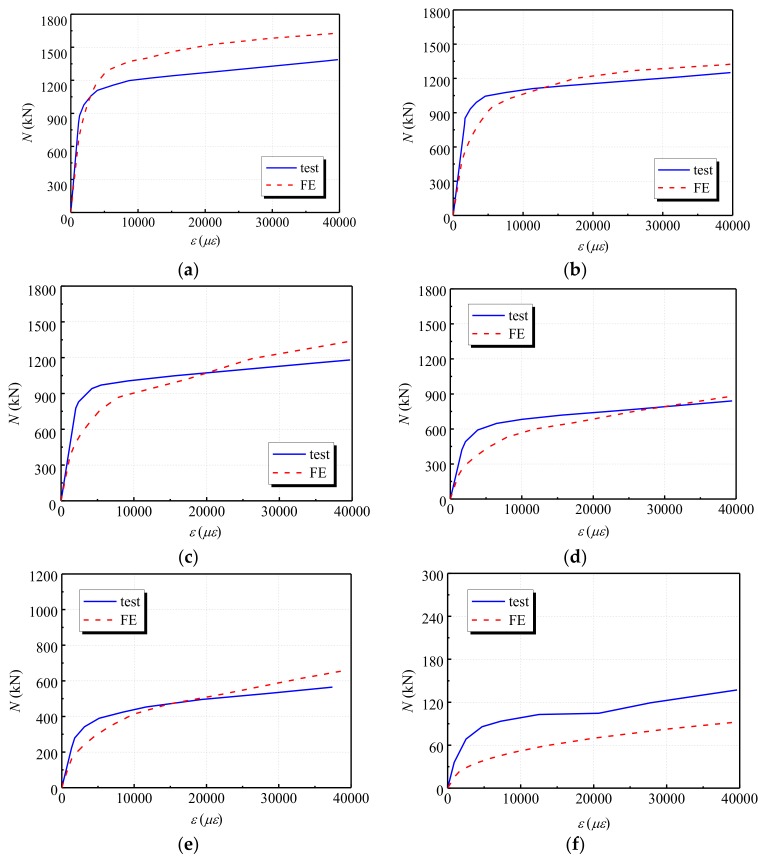
Load-strain curve comparisons between experimental and numerical results (**a**); SC-1 (**b**) SC-2; (**c**) SC-3; (**d**) SC-4; (**e**) SC-5; (**f**) SC-6.

To further validate the capacity of the developed finite element (FE) model, a comparative study was conducted by comparing the experimental results in [30] with the corresponding numerical results from the FE model in this study. The mechanical performances of six circular steel tubular short column specimens at 20 °C, 200 °C, 300 °C, 500 °C, 600 °C and 900 °C temperatures in ref. [30] were calculated using the developed model. Table 6 provides information on the parameters of six specimens in ref. [30]. Then, the simulation results were compared to the experimental results, which are shown in Figure 10. It can be seen from the figure that the simulation results match the testing results very well, which indicates the effectiveness of the FE model.

### 3.3. Mechanism Analysis

#### 3.3.1. Stress Analysis of Concrete

**Figure 11:**
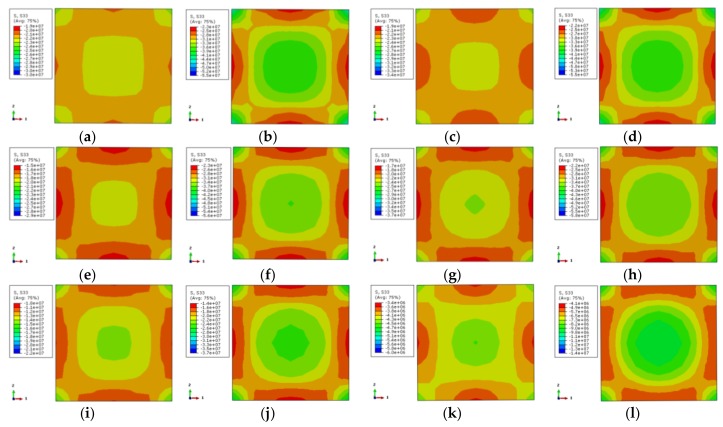
Stress distribution of concrete in the middle cross section of columns (**a**) DS-20-0.75; (**b**) DS-20-1; (**c**) DS-100-0.75; (**d**) DS-100-1; (**e**) DS-200-0.75; (**f**) DS-200-1; (**g**) DS-400-0.75; (**h**) DS-400-1; (**i**) DS-600-0.75; (**j**) DS-600-1; (**k**) DS-800-0.75; (**l**) DS-800-1.

The longitudinal stress distribution of the concrete is shown in Figure 11. The stress contour corresponds to 0.75 times the ultimate bearing capacity at the rising-stage of the longitudinal load–displacement curve of the specimen. The longitudinal stress distribution of two characteristic points on the longitudinal load–displacement curves is symmetric, where the corner area of the section is a high-stress zone. The core region of the section takes second place while the middle region of the column is the lowest. This indicates that the concrete in the corner is restrained by the steel tube, and the concrete at the core area is restrained by the external concrete, where the steel tube has a weak restraint effect. Although the middle area of the column is restrained by stiffening ribs and steel tube, its stress is lower. When the characteristic point of the load–displacement curve achieves 0.75 times the ultimate bearing capacity, the longitudinal stress gradient of the cross-section is less. The load increases to the ultimate bearing capacity, and the longitudinal stress gradient of the cross-section increases as well. Among them, the stress at the external corner and the core region of the cross-section increases while the stress in the middle of the column is not obvious. With the increase of temperature, the stress gradient of the concrete is increased, and the stress value is decreased.

**Figure 12:**
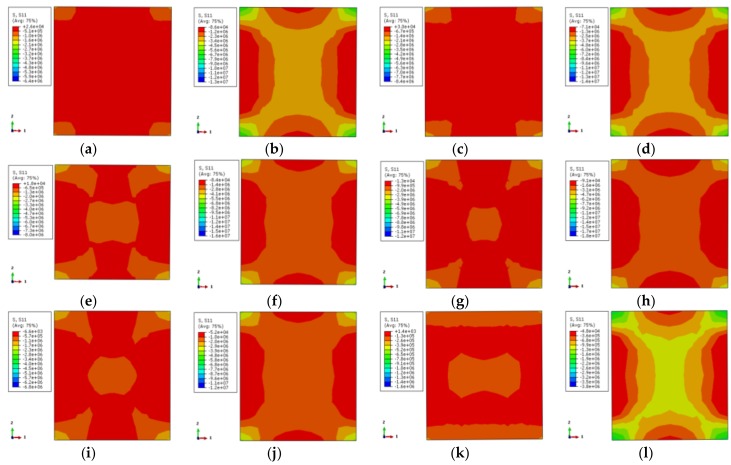
The lateral stress distribution of concrete (**a**) DS-20-0.75; (**b**) DS-20-1; (**c**) DS-100-0.75; (**d**) DS-100-1; (**e**) DS-200-0.75; (**f**) DS-200-1; (**g**) DS-400-0.75; (**h**) DS-400-1; (**i**) DS-600-0.75; (**j**) DS-600-1; (**k**) DS-800-0.75; (**l**) DS-800-1.

The transverse stress distribution of the concrete (one-direction) at elevated temperature is illustrated in Figure 12. The transverse stress distribution of the two characteristic points in the load–displacement curve is symmetric. The external corner is the high-stress zone followed by the core area, while the middle region of the column is the low-stress area. When the characteristic point achieves 0.75 times the ultimate bearing capacity, there is a large area of low-stress at ambient temperature and elevated temperature of 100 °C, except for the small area of the corner. With the increase of temperature, the core region and the corner become the high-stress zone while other areas are low-stress areas. When the characteristic point achieves the ultimate bearing capacity, the high-stress zone (X-shape) occurs in the adjacent diagonal area and the core region for the specimens occurs at 100 °C temperature and ambient temperature. The stress in the diagonal and core regions declines with the increase of temperature. The stress gradient of specimens under 800 °C temperature increases obviously, however, the distribution is basically the same.

#### 3.3.2. Influence of Reinforcing Ribs

To analyze the influence of reinforcing ribs on the axial compression performance of the specimen, the specimens DS-200-0/DS-200 and DS-600-0/DS-600 in this paper were selected. The stress distributions of the stiffening ribs, concrete, and steel tube were analyzed.

**Figure 13:**
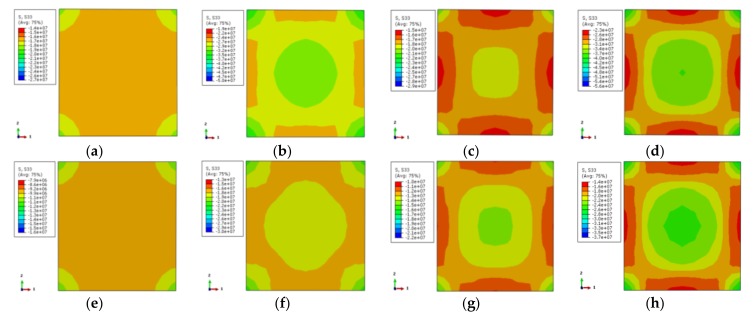
The vertical stress distribution of concrete (**a**) DS-200-0-0.75; (**b**) DS-200-0-1; (**c**) DS-200-0.75; (**d**) DS-200-1; (**e**) DS-600-0-0.75; (**f**) DS-600-0-1; (**g**) DS-600-0.75; (**h**) DS-600-1.

In Figure 13, the longitudinal stress distributions of all specimens are uniform and comply with the temperature in Figure 11. When the characteristic point of the load–displacement curve corresponds to 0.75 times of the ultimate bearing capacity, the longitudinal stress of the non-stiffened specimen is relatively small except in the corner area, and the stress gradient is also less. After setting the reinforcement rib, the longitudinal stress in the center area of the cross-section increases and the stress gradient of the cross-section increases as well. The overall stress distribution tends to be homogeneous. When the characteristic points correspond to the ultimate bearing capacity, the longitudinal stress is the largest at the external corner of the non-stiffened specimen, followed by the center area, and the longitudinal stress at the rest area is small. After setting the reinforcement rib, the high-stress region of the center of the cross-section is enlarged, and the stress gradient is increased. The overall trend of stress distribution becomes uniform.

**Figure 14:**
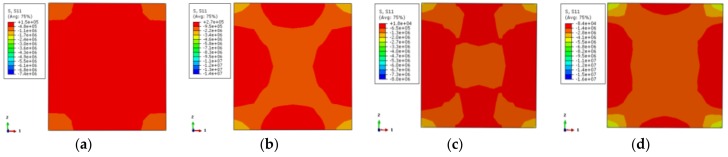
The lateral stress distribution of concrete (**a**) DS-200-0-0.75; (**b**) DS-200-0-1; (**c**) DS-200-0.75; (**d**) DS-200-1.

Figure 14 shows the transverse stress distribution (one-direction) of the concrete with the stiffener at 200 °C temperature. It corresponds to 0.75 times the ultimate bearing capacity in the rising-stage of the longitudinal load–displacement curve of the specimen. The transverse stress distribution of the stiffened rib corresponding to the characteristic point in the load–displacement curve of the case of 200 °C is symmetrical. The external corner of the specimen is high-stress zone while the middle area of the column is low-stress zone. When the characteristic point is 0.75 times the ultimate bearing capacity, the stress level in most areas is small, except for that in the corner. The overall stress contour of the section is in a petal shape after the reinforcement ribs are set for the stiffener enhances the stress. When the characteristic point achieves the ultimate bearing capacity, the stress distributions of the specimen cross-section are the same. The stress in the corner is enhanced, and the low-stress zone in the central area of the column is reduced.

**Figure 15:**
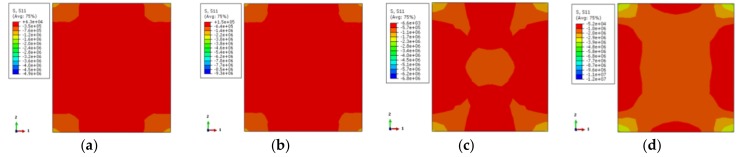
The lateral stress distribution of concrete (**a**) DS-600-0-0.75 (**b**) DS-600-0-1 (**c**) DS-600-0.75 (**d**) DS-600-1.

Figure 15 shows the contour of the transverse stress distribution (one-direction) of the concrete with the stiffened rib at 600 °C temperature. The overall trend of stress distribution is basically consistent with that of the specimen at 200 °C temperature. The reductions of the mechanical properties of the specimen are severe because of the elevated temperature, which contributes to obvious stress level reduction.

**Figure 16:**
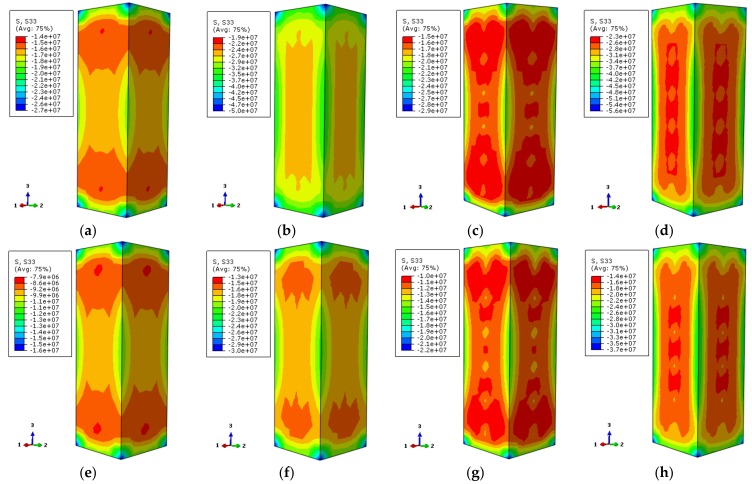
The vertical stress distribution of concrete (**a**) DS-200-0-0.75; (**b**) DS-200-0-1; (**c**) DS-200-0.75; (**d**) DS-200-1; (**e**) DS-600-0-0.75; (**f**) DS-600-0-1; (**g**) DS-600-0.75; (**h**) DS-600-1.

Figure 16 depicts the longitudinal stress distribution of the concrete along the column height corresponding to 0.75 times the ultimate bearing capacity and ultimate bearing capacity of the load–displacement curve, respectively. The stress of the column is the largest at both top and bottom locations of the column. In addition, due to the restrained effect on the steel tube, the stress is larger at the external corner. Non-stiffened specimens have less stress near the ends of the column at one-fourth of the location. With the load increasing to the ultimate bearing capacity, the overall distribution of longitudinal stress of the specimen is basically the same. The stress distribution of the rebar stiffened specimen tends to be uniform. This shows that the mechanical properties can be improved by setting reinforcement ribs for stress redistribution.

**Figure 17:**
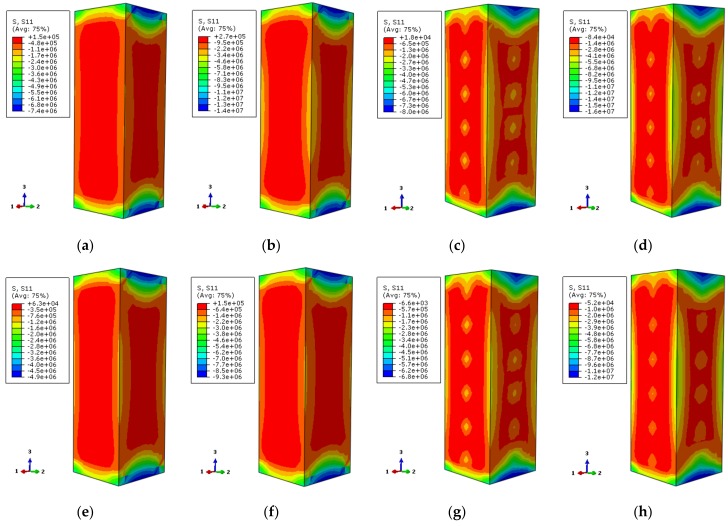
The lateral stress distribution of concrete (**a**) DS-200-0-0.75; (**b**) DS-200-0-1; (**c**) DS-200-0.75; (**d**) DS-200-1; (**e**) DS-600-0-0.75; (**f**) DS-600-0-1; (**g**) DS-600-0.75; (**h**) DS-600-1.

Figure 17 illustrates the transverse stress (one-direction) distribution of the concrete along the column height corresponding to 0.75 times the ultimate bearing capacity and ultimate bearing capacity on the load–displacement curve, respectively. The transverse stress of the non-stiffened specimen displays a large range of low-stress zone. When the load increases to the ultimate bearing capacity, the lower-stress zone is reduced because of the external corner restriction. The stress distribution of specimens with reinforced stiffeners is almost consistent. Compared with the non-stiffened specimen, the high-stress zone of the external corner of the column is a little bit larger and the stress value near the stiffened rib has an obvious increase. The overall stress distribution of the specimen tends to be homogeneous owing to the fact that the steel tube of the stiffened specimen plays a better restraining role.

**Figure 18:**
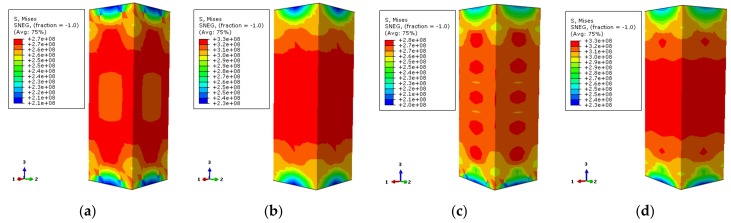
The Mises stress distribution of the steel tube (**a**) DS-200-0-0.75; (**b**) DS-200-0-1; (**c**) DS-200-0.75; (**d**) DS-200-1.

**Figure 19:**
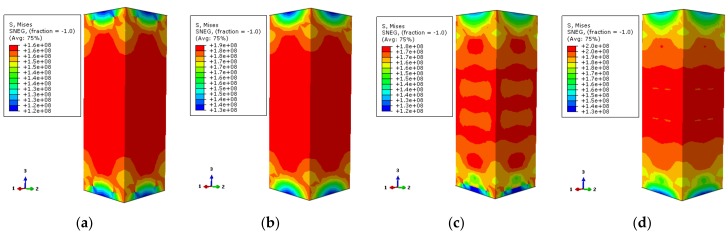
The Mises stress distribution of the steel tube (**a**) DS-600-0-0.75 (**b**) DS-600-0-1 (**c**) DS-600-0.75 (**d**) DS-600-1.

The contours of Mises stress distribution along the column height direction by finite element numerical calculation are shown in Figure 18 and Figure 19. The selected characteristic points correspond to 0.75 times of the ultimate bearing capacity and ultimate bearing capacity of the load–displacement relation curve of the specimen, respectively.

In Figure 18, the stress values of the stiffened and non-stiffened columns are extracted from the high-stress zone of steel tube at 200 °C temperature, respectively. When the load–displacement curve of the non-stiffened specimen corresponds to 0.75 times of the ultimate bearing capacity, the stress value of the high-stress location of the steel tube is about 270 MPa. The stress value is about 325 MPa when the ultimate bearing capacity is reached. For the stiffened specimen, the stress value of the steel tube is about 275 MPa in the rising stage of the load–displacement curve. It is about 324 MPa when the ultimate bearing capacity is reached. Referring to the yield strength of the steel at high temperature, the study does not consider the reduction of the steel strength at 200 °C temperature, which is 338.5 MPa. At this moment, the stress value of the steel tube in the high-stress region does not reach its yield strength for both stiffened and non-stiffened specimens. When the ultimate bearing capacity is reached, the stress values of stiffened and non-stiffened specimens are close to the yield strengths but do not reach the yield strengths. There is a small difference of the stress value between stiffened specimen and non-stiffened specimen. However, after setting the stiffening rib, the steel tube has a more uniform constraint influence on the concrete so that the axial compressive performance of the specimen is improved.

In Figure 19, the stress values of the stiffened and non-stiffened specimens are extracted in the high-stress zone of the steel tube at 600 °C temperature, respectively. When the load–displacement curve of the non-stiffened specimen corresponds to 0.75 times of the ultimate bearing capacity in the rising stage, the stress value of the high-stress zone of the steel tube is about 163 MPa. When the ultimate bearing capacity is reached, the stress value is about 189 MPa. The stress value of the steel tube is about 155 MPa when the load–displacement curve of the stiffened specimen corresponds to 0.75 times the ultimate bearing capacity in the rising stage. The stress value is about 180 MPa when the ultimate bearing capacity is reached. Based on the yield strength of the steel under high temperature, for instance, it is 152 MPa at 600 °C temperature. The load–displacement curve of the specimen corresponds to 0.75 times of the ultimate bearing capacity and ultimate bearing capacity in the rising stage. The stress values of the stiffened specimen and non-stiffened specimen reach their yield strengths. There are large thermal expansion and axial compression deformation of the materials in the specimen at high temperature. The yield strength of the external steel tube can be reached. Good integrality can be maintained after the stiffened specimen fails.

**Figure 20:**
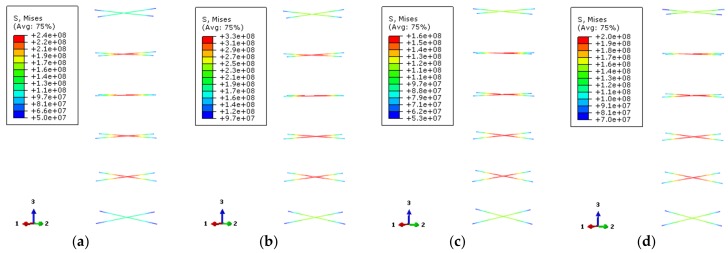
The Mises stress distribution of bar stiffeners (**a**) DS-200-0.75; (**b**) DS-200-1; (**c**) DS-600-0.75; (**d**) DS-600-1.

Figure 20 provides the Mises stress distributions of the stiffened ribs (Mises stress of rebar stiffener is its tensile stress). The selected characteristic points correspond to 0.75 times of the ultimate bearing capacity and ultimate bearing capacity of the load–displacement curve of the specimen, respectively. The stress of the stiffened rib near the middle of the column is the largest and decreases at both ends of the column.

According to the yield strength of the steel at high-temperature, the corresponding values should be 376.7 MPa and 170 MPa at 200 °C and 600 °C temperatures, respectively. When the load–displacement relationship curve of the specimen at 200 °C temperature corresponds to 0.75 times of the ultimate bearing capacity and the ultimate bearing capacity, the stress values of reinforced stiffener are about 227 MPa and 327 MPa, respectively. For the case of 600 °C temperature, the stress values are about 125 MPa and 163 MPa, respectively. The stiffening rib of the specimen at 200 °C temperature does not reach its yield strength at the two characteristic points of the load–displacement curve. At 600 °C high temperature, its yield strength is close to the ultimate bearing capacity, which indicates that the transverse deformation of the reinforced ribs near the yield location of the steel tube is larger.

#### 3.3.3. Interaction between Concrete and Steel Tubular

**Figure 21:**
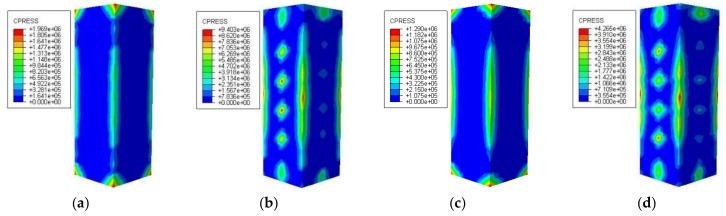
Distributions of the restraining force of steel tube to concrete (**a**) DS-200-REF; (**b**) DS-200; (**c**) DS-600-REF; (**d**) DS-600.

Figure 21 shows the restraining force distribution of the steel tube to the concrete when the loading of the specimen reaches the peak load. It can be seen that under the high temperature conditions of 200 °C and 600 °C, the restraining force distribution of the steel tube to the concrete is consistent, and the restraining force of the steel stiffening specimen is concentrated in the vicinity of the corner of the test specimen and the stiffened steel bar, wherein the restraining force in the adjacent area of the stiffened steel bar is better. The restraining force of the non-stiffened specimen is concentrated at the corners of the specimen. It can be seen that for the steel bar stiffened specimen, the larger the area of the steel tube restraining the concrete, the better the restraining effect.

**Figure 22:**
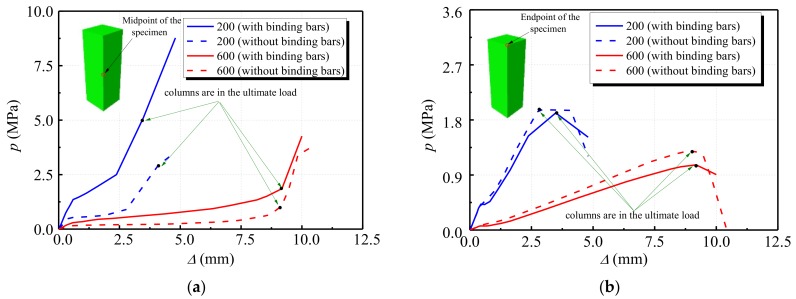
Relationship between restraining force and axial displacement (**a**) middle of column; (**b**) top of column.

Figure 22 shows the change relationship between the restraining force at the corners of the steel tube and the axial displacement of cross-sections in the middle and top areas of the column, respectively. It can be seen from Figure 22a that the restraining force of the corner of the stiffened specimen under constant temperature of 200 °C is higher than that of the non-stiffened specimen. The restraining forces of both of them are accelerated when they reach about 0.68 times of the peak displacement. When the load of the specimen at the temperature of 600 °C reaches the peak displacement, the growth rate of the restraining force increases. Compared with the specimen at the temperature of 200 °C, the specimen at the temperature of 600 °C has less restraining force and the growth becomes slower. It can be seen from Figure 22b that the restraining force at the corner of the stiffened specimen at the same temperature is slightly lower than that of the non-stiffened specimen. When both types of specimen reach the peak displacement, the restraining force starts to decrease, and the restraining force of the non-stiffened specimen decreases faster. Among them, when the non-stiffened specimen reaches the peak displacement of 1.15 times at 600 °C, the steel tube at the end of the column is completely destroyed. It can be seen that for the steel bar, stiffened and non-stiffened specimens, the restraining effect of the steel tube in the middle of the column is better than that of the steel tube at the corner of the column.

## 4. Conclusions

Based on the experiment and the numerical analysis results in this study, the detailed conclusions can be drawn as follows.

(1) The buckling curvatures of the steel tube are in the middle of the column and the adjacent areas, the locations of which are not the same on different sides. The buckling curvature of the non-stiffened specimen is higher than that of the stiffened specimen. There is no obvious damage of the concrete, except the damage on the opposite sides of the steel tube buckling curvature position of the oblique line, which forms the shear surface. Consequently, the failure of each specimen is shear damage.
(2) Residual bearing capacity and compressive stiffness of the test specimen decreases significantly with the increase of the temperature. Compared with the specimen under normal temperature, the ultimate bearing capacities of the specimens at 100 °C, 200 °C, 400 °C, 600 °C, and 800 °C decrease by 7.72%, 12.3%, 17.64%, 35.55%, and 75.83%, and the compressive stiffness values decrease by 11.79%, 14.83%, 63.12%, 72.24%, and 90.49%, respectively. Among them, when the temperature exceeds 200 °C, the mechanical properties of the specimen begin to be greatly reduced, the ultimate bearing capacity decreases rapidly, and the ductility coefficient decreases with the increase of the temperature. The addition of stiffened steel bars has a positive impact on improving the mechanical properties of concrete filled steel tubes. The critical temperature of the test specimen is between 200 °C and 400 °C. It shows that the decrease rates of residual bearing capacity and compressive stiffness of the specimen increase after the temperature exceeds 200 °C. This is mainly because the concrete strength decreases rapidly after the furnace temperature exceeds the critical temperature.
(3) Using the finite element software ABAQUS, the model of thin-walled square steel tubular short column with stiffening bars under constant temperature is established. The stress distribution of the concrete, steel tube, and reinforcing rib of the specimen were analyzed. With the increase of temperature, the longitudinal stress gradient of the concrete increases while the stress value decreases. Compared with non-stiffened specimens, the longitudinal stress in the center of the section increases, the stress gradient increases, and the overall distribution tends to be homogeneous. The transverse restraint of the stiffener can restrict the outer buckling of the steel tube and change the buckling mode. The stress distribution of the steel plate becomes relatively uniform and the restraint effect of the concrete is enhanced due to setting of the reinforcing rib.

This work is only based on plain CFST. In future work, a novel CFST with new composites will be developed and the effect of high-temperature on the performance of the composite structures will be further investigated for practical application. Besides, the fracture shape of the specimen under high-temperature exposure will be investigated and compared with the simulation results to further validate the effectiveness of the developed numerical model.

## Figures and Tables

**Table 1:** Main parameters of specimens.

Specimen	*B* × *t*_s_ × *H* (mm × mm × mm)	Diameter of Stiffening Bar *d* × Spacing (mm × mm)	Temperature (°C)
DS-20	160 × 2 × 480	6 × 80	20 (Ambient Temperature)
DS-100	160 × 2 × 480	6 × 80	100
DS-200-0	160 × 2 × 480	none	200
DS-200	160 × 2 × 480	6 × 80	200
DS-400	160 × 2 × 480	6 × 80	400
DS-600-0	160 × 2 × 480	none	600
DS-600	160 × 2 × 480	6 × 80	600
DS-800	160 × 2 × 480	6 × 80	800

^a^ Named specimen as follows: DS denotes square column, the first number represents heated temperature (°C), and the second number represents whether or not stiffening column.

**Table 2:** Mechanical properties of steel.

Steel Types	*f*_y_ (Mpa)	*f*_u_ (Mpa)	*E*_s_ (Mpa)	Elongation *δ*_10_ (%)
Steel plate (*t*_s_ = 2 mm)	338.5	451.3	197600	38.79
Steel bar (*d*_s_ = 6 mm)	376.7	467.8	184300	26.7

**Table 3:** Mechanical properties of concrete.

*F_cu_*_,28_/(Mpa)	*f_cu,test_* (Mpa)	*E_c_*_,28_ (× 10^4^ Mpa)	*E_c_*,_*test*_ (× 10^4^ Mpa)	Concrete Age (Day)
32.3	41.9	2.241	3.527	97

**Table 4:** Specimen heating parameters.

Specimen	DS-20	DS-100	DS-200-0	DS-200	DS-400	DS-600-0	DS-600	DS-800
Temperature (°C)	20	100	200	200	400	600	600	800
Time (min)	0	1	2.5	2.5	5	7.5	7.5	10

**Table 5:** Testing results.

Specimen	*E*A (10^6^ kN)	*N*_y_ (kN)	*△*_y_ (mm)	*N*_u_ (kN)	*△*_u_ (mm)	*△*_0.85_ (mm)	*μ*_△_
DS-20	0.789	1079.487	1.763	1177.53	2.92	3.62	1.24
DS-100	0.696	951.060	2.208	1086.63	3.15	4.91	1.56
DS-200	0.672	858.748	2.725	1032.73	5.46	7.63	1.40
DS-400	0.291	763.468	6.822	969.78	12.61	15.93	1.26
DS-600	0.219	568.282	8.901	758.89	14.59	19.79	1.36
DS-800	0.075	215.331	9.303	284.62	15.85	21.25	1.34
DS-200-0	0.675	847.427	2.749	1050.72	4.15	6.06	1.46
DS-600-0	0.221	523.305	7.494	695.92	12.87	17.22	1.34

**Table 6:** The parameters of the specimens in ref. [30].

Specimen	*D* (mm)	*t* (mm)	*f_cu_* (MPa)	*f_y_* (MPa)	Temperature (°C)
SC-1	133	4.5	40.8	324	20
SC-2	133	4.5	40.8	324	200
SC-3	133	4.5	40.8	324	300
SC-4	133	4.5	40.8	324	500
SC-5	133	4.5	40.8	324	600
SC-6	133	4.5	40.8	324	900
